# Washed microbiota transplantation improves sleep quality in patients with sleep disorder by the gut-brain axis

**DOI:** 10.3389/fnins.2024.1415167

**Published:** 2024-06-24

**Authors:** Hongxin He, Manqing Li, Yifan Qiu, Zhiqing Wu, Lei Wu

**Affiliations:** ^1^Sun Yat-sen University School of Medicine, Guangzhou, China; ^2^Department of Gastroenterology, Research Center for Engineering Techniques of Microbiota-Targeted Therapies of Guangdong Province, The First Affiliated Hospital of Guangdong Pharmaceutical University, Guangzhou, China; ^3^Guangzhou Xinhai Hospital, Guangzhou, China; ^4^School of Biological Sciences and Engineering, South China University of Technology, Guangzhou, China

**Keywords:** fecal microbiota transplantation, washed microbiota transplantation, sleep disorder, sleep quality, life quality

## Abstract

**Background:**

The clinical impact of washed microbiota transplantation (WMT) from healthy donors in sleep disorder (SD) patients is unclear. This study aimed to investigate the effect of WMT in SD patients.

**Methods:**

The clinical data were collected from patients with different indications receiving 1–3 courses of WMT, divided into two groups by 7 points of PSQI scale. The score of PQSI and SF-36 scale was used to assess the improvement in sleep quality and life quality among patients with sleep disorders following WMT. Finally, 16S rRNA gene amplicon sequencing was performed on fecal samples of patients with sleep disorders before and after WMT.

**Results:**

WMT significantly improved sleep quality in patients with sleep disorder in the short and medium term. WMT significantly improved sleep latency, sleep time and total score in the short term. WMT significantly improved sleep quality and total score in the medium term. In terms of sleep quality and sleep latency, the improvement value also increased with the increase of treatment course, and the improvement effect of multiple treatment course was better than that of single and double treatment course. In the total score, the improvement effect of double and multiple treatment was better than that of single treatment. WMT also improved quality of life in the sleep disorder group. WMT significantly improved general health, vitality, social function and mental health in the short term. WMT significantly improved role-physical, general health, vitality, and mental health in the medium term. WMT regulated the disturbed gut microbiota in patients with sleep disorders. In the normal sleep group, WMT had no effect on the decline of sleep quality in the short, medium and long term, and had an improving effect on the quality of life.

**Conclusion:**

WMT could significantly improve sleep quality and life quality in patients with sleep disorders with no adverse events. The improvement in sleep quality resulting from WMT could lead to an overall enhancement in life quality. WMT could be a potentially effective treatment for patients with sleep disorders by regulating the gut microbiota.

## Introduction

Sleep is the most basic physiological activity of human beings. Healthy sleep is essential for brain development, immune function, and metabolism. During the novel COVID-19 pandemic, the global prevalence of sleep disorders (SD) was about 35% ± 5% (Jahrami et al., 2021, 2022; Alimoradi et al., 2022). A variety of factors such as stress, anxiety, and depression can cause SD, which has made SD an increasing concern. Studies have shown that SD was closely related to the cardiovascular, metabolic, and nervous systems (Altevogt and Colten, 2006; Neroni et al., 2021). In addition, lack of sleep has been linked to digestive disorders. Studies have found that sleep deprivation leaded to the accumulation of reactive oxygen species (ROS) in the gut, leading to many inflammatory bowel diseases and even premature death (Fass et al., 2000; Kloska et al., 2020; Vaccaro et al., 2020).

In clinical practice, there were several common approaches to improving sleep quality. First, medication was commonly used to treat sleep disorders; however, it often caused side effects like drowsiness, impaired movement, and the need for increased dosage over time. Second, lifestyle changed such as dietary adjustments and improvements in the sleep environment may help alleviate sleep disorders, although they may not be sufficient for treating all cases. Third, non-medical techniques like Cognitive Behavioral Therapy for Insomnia (CBT-I) have been developed to address negative thought patterns and behaviors. Despite these approaches, sleep disorders remain a challenging issue, indicating a need for comprehensive solutions. Recent research suggested that the gut microbiota could influence sleep disorders through the entero-cerebral axis, highlighting the potential value of adjusting the gut microbiota for improving sleep. Fecal microbiota transplantation (FMT) was a novel treatment that used a healthy microbial configuration to replace the patient’s own disturbed microbiota (Bafeta et al., 2017). FMT has the tendency not only to improve the function of symbiotic host bacteria, but also to completely reshape the entire host microbiome. FMT accomplished this by changing the actual composition and proportion of resident symbiotic species present in the host. FMT was included in guidelines that recommend its used as the standard of care in cases of recurrent *Clostridium difficile* infection (Varier et al., 2015). FMT was gaining increasing interest (de Groot et al., 2020). FMT was being tested in clinical trials for other diseases, such as inflammatory bowel disease (IBD) (Moayyedi et al., 2015; Paramsothy et al., 2017; Qazi et al., 2017), Crohn’s disease (Paramsothy et al., 2017), obesogenic obesity (Allegretti et al., 2020), and functional gastrointestinal disorders (Pamer, 2014), which were also associated with significant ecological disorders. Whether FMT can improve SD remains to be discussed in clinical medicine.

Washed microbiota transplantation (WMT) was similar to traditional FMT, but WMT has better safety, quality control, and effectiveness against diseases with flora disorders (Zhang et al., 2020). Due to its ability to alter the gut microbiota, WMT has the potential to ameliorate neuropathic diseases through the gut-brain axis, which allows bidirectional communication between the gut and the brain. We attempted to investigate the patients who received WMT treatment in the Department of Gastroenterology of the First Affiliated Hospital of Guangdong Pharmaceutical University for other diseases, to observe whether WMT has an improvement effect on sleep quality in patients with SD. We hypothesized that WMT can safely and consistently improve patients with SD without adverse events. This study aimed to investigate the effects of WMT on sleep quality in patients with SD.

## Materials and methods

### Preparation of washed microbiota and WMT procedure

Washed microbiota transplantation (WMT) was a method aimed at reconstructing the gut microbiota of individuals by screening, preparing, and transplanting washed microbiota from healthy donors to patients. The screening process for selecting suitable donors, preparing eligible suspensions for medication in the clinic, and conducting transplantation under an endoscope adhere to the Nanjing consensus on methodology of washed microbiota transplantation (Zhang, 2020). This study recommended patients to follow the “thrice thrice therapy” for receiving WMT treatment and consolidation treatment. The concept of “thrice thrice therapy”: in the first three months after starting WMT treatment, the patients should undergo WMT treatment once a month (injecting washed microbiota suspension for three consecutive days each month). After that, there should be a three-month interval before the fourth WMT treatment (five months after baseline), in order to strengthen the colonization of transplanted microbiota through this treatment. However, due to different levels of compliance from patients, not all of them can complete the WMT treatment as scheduled.

### Patients and study design

This study aimed to assess the efficacy of WMT in improving sleep quality and life quality among patients with sleep disorders, who received WMT treatment for their medical condition at The First Affiliated Hospital of Guangdong Pharmaceutical University between October 18th, 2020, and October 30th, 2022. Inclusion criteria: only patients who had at least one follow-up visit, were between the ages of 18 and 80, and did not use sedation hypnosis in the previous year were included in the study. Exclusion criteria: pregnant women, patients taking antibiotics, hormones and probiotics during the first 3 months of WMT and during transplantation. Patients with factors that could negatively impact data collection, such as inability to express their own status or cooperate with the questionnaire survey (e.g., patients with mental disorders or autism), were excluded.

The Pittsburgh Sleep Quality Index (PSQI) scale was used to evaluate the sleep quality of the patients. The scale consists of 19 self-rated items and 5 other-rated items. The 19th self-rated item and the 5 other-rated items were not included in the scoring. The 18 self-rated items that were included in the scoring process were combined into 7 components that determine the sleep quality index: sleep quality, sleep latency, sleep time, sleep efficiency, sleep disorders, use of hypnotic drugs, and daytime dysfunction. Each component was scored from 0 to 3 points. The total PSQI score was the sum of the scores for the 7 components, ranging from 0 to 21. A higher score indicates poorer sleep quality.

The 36-item Short Form Health Survey scale (SF-36) was used to evaluate the quality of life of the patients. The SF-36 health survey short form contains 8 dimensions, including physical functioning (PF), role-physical (RP), bodily pain (BP), general health (GH), vitality (VT), social functioning (SF), role-emotional (RE), and mental health (MH). This scale consists of a total of 36 items. The initial scores of the 8 dimensions were calculated by summing the scores of each item within the dimension. These initial scores were then converted into final scores on a scale of 0 to 100 to allow for comparison between the dimensions. The conversion formula used was: final score = (actual initial score - theoretical minimum initial score) / (theoretical highest initial score - theoretical minimum initial score) × 100. A higher score indicated a better quality of life.

To establish the baseline values, we collected PQSI and SF-36 scale data from the patients during their initial course of treatment. These baseline values were then compared with subsequent scale scores obtained at each subsequent course or follow-up session. Each enrolled patient could contribute data for up to four courses, including the baseline value, the value after the first course of WMT treatment (short-term), the value after the second course (medium-term), and the value after the third course (long-term).

### Clinical data collection

The study included 63 patients, and the Pittsburgh Sleep Quality Index (PSQI), developed by Buysee et al., was used to assess sleep quality. In accordance with the standards set by domestic sleep research, a PSQI score of 7 was used as the benchmark for assessing sleep quality (Buysse et al., 1989). Participants with a total score greater than 7 points were assigned to the sleep disorder group (*n* = 40). Scores between 8 and 16 were classified as mild to moderate sleep disorder, while scores of 17 or higher were categorized as severe sleep disorder, indicating worse sleep quality. Participants with a total score equal to or less than 7 were allocated to the normal sleep group (*n* = 23), denoting better sleep quality with lower scores.

We obtained data on age, gender, reasons for undergoing WMT, treatment duration, and associated adverse events. At each treatment, we collected scale scores that represented treatment efficacy. Additionally, patients were categorized into three groups based on the number of WMT treatments: the short-term treatment group (compared the first WMT session with the baseline), the medium-term treatment group (compared the second WMT session with the baseline), and the long-term treatment group (compared the third WMT session with the baseline).

### DNA extraction and sequencing

Stool samples were collected from 8 SD patients before WMT, 8 SD patients after WMT (short-term), and 5 donors for sequencing. All samples were stored at −80°C after collection until DNA extraction. Microbial DNA was extracted using the QIAamp DNA stool mini kit (QIAGEN, Hilden, Germany). DNA quality and concentration were examined by NanoDrop^™^ 2000 (Thermo Fisher Scientific, Wilmington, DE, United States). Primers 338F and 806R were used for PCR amplification of bacterial 16S rRNA gene fragments (V3-V4) from extracted DNA. After qualified library, NovaSeq6000 (Illumina, San Diego, CA, United States) sequencing platform was used for machine sequencing.

### Amplicon data processing and analysis

From all the sample data of splited out of a plane data, amputation of barcode and primer sequences after use FLASH (V1.2.11^1^) (Magoč and Salzberg, 2011) software to splice the sample reads, get Raw Tags. Then fastp (0.19.6) (Chen et al., 2018) software was used to conduct quality control on the obtained Raw Tags, and high-quality Clean Tags were obtained. Finally, Clean Tags were compared with the database to detect and remove chimeras (Haas et al., 2011), so as to obtain the Effective Tags. The DADA2 Variants in QIIME2 (version 2020.2) were used and sequences with abundance less than 5 were filtered out to obtain the final ASVs variants and feature lists for the variants. The resulting ASVs were then compared with the database using the classify-sklearn module in QIIME2 software to get species information for each ASV.

### Statistical analysis

This study employed SPSS 22.0 (IBM Corp., Armonk, NY, United States) and Prism 8 (GraphPad, San Diego, CA, USA) for statistical analysis. For normally distributed continuous variables, the results were expressed as mean and standard deviation. An unpaired Student’s *t*-test (for normally distributed variables) could be used for comparison of continuous variables between two independent groups. For categorical variables, the results were presented as frequencies and percentages, and analyzed using chi-square or Fisher’s exact test. Paired data were compared using paired Student’s *t*-test (for normally distributed variables). A two-tailed *p*-value less than 0.05 was considered statistically significant.

## Results

### Clinical features of patients receiving WMT

**Figure 1:**
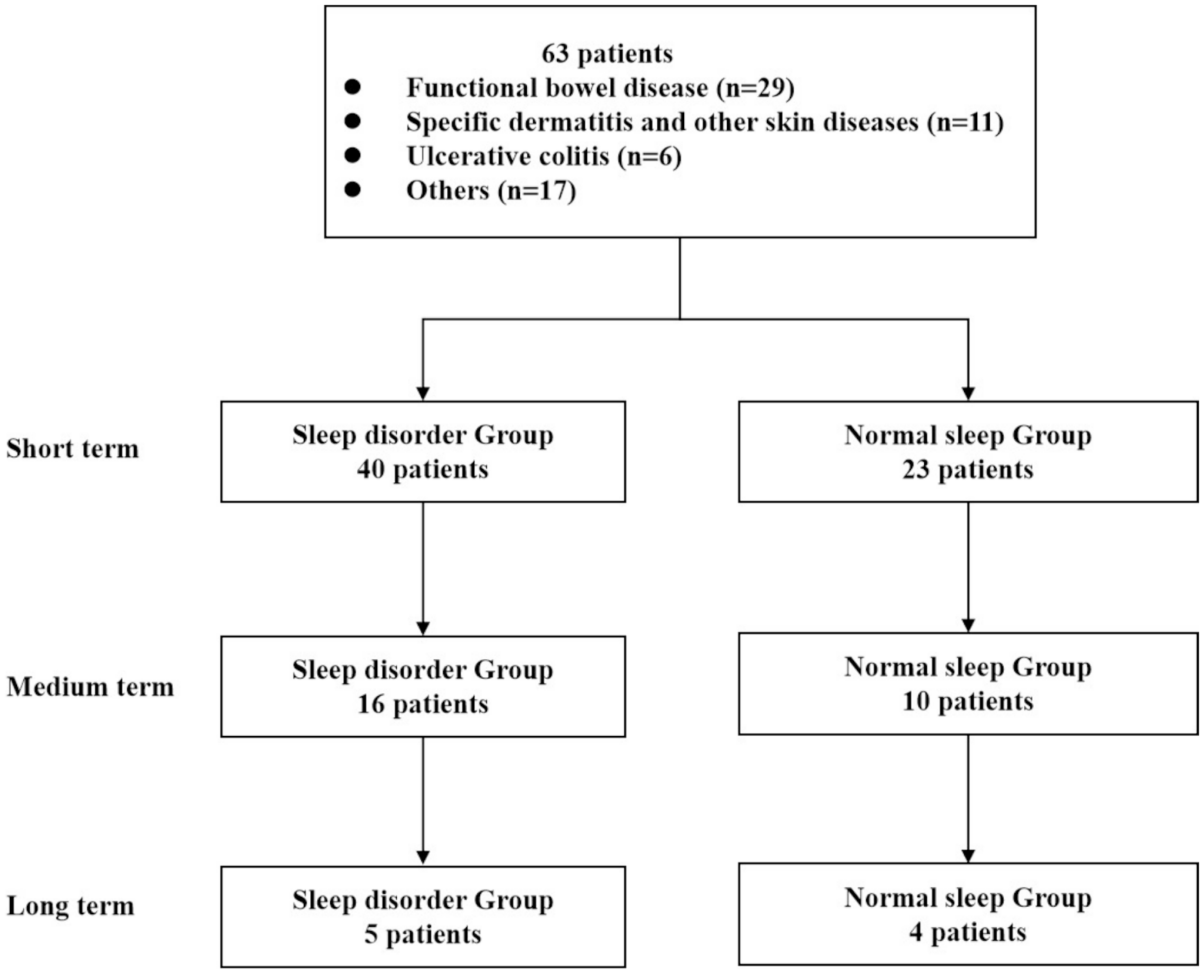
Flow chart of this study.

According to the inclusion and exclusion criteria, this study included a total of 63 patients’ relevant information for statistical analysis. Among them, there were 35 male patients (accounting for 55.6%) and 28 female patients (accounting for 44.4%). The average age was 45.79 ± 15.94 years, with a minimum age of 19 years and a maximum age of 78 years. Statistical analysis revealed that the top six causes of WMT treatment among the collected samples were functional bowel disease (29 individuals, 46.03%), specific dermatitis and other skin diseases (11 individuals, 17.46%), ulcerative colitis (6 individuals, 9.52%), hyperlipidemia (5 individuals, 7.94%), chemotherapy-induced diarrhea (3 individuals, 4.76%), and gastroesophageal reflux disease (3 individuals, 4.76%) (Figure 1). Please refer to Table 1 for more details. Now, the time intervals for WMT treatment among the included patients were analyzed and expressed in days using the median value (25–75%). The baseline value was determined by the first set of questionnaire data completed during the first treatment course. The second treatment course had a short-term interval from the baseline [34 (30–40) days], the third treatment course had a medium-term interval from the baseline [74 (62–84) days], and the fourth treatment course had a long-term interval from the baseline [160 (137–231) days].

**Table 1:** The main diagnoses of patients receiving washed microbiota transplantation.

Cause of WMT	Number	Percentage
Functional bowel disease	29	46.03%
Specific dermatitis and other skin diseases	11	17.46%
Ulcerative colitis	6	9.52%
Hyperlipidemia	5	7.94%
Chemotherapy-induced diarrhea	3	4.76%
Gastroesophageal reflux disease	3	4.76%
Radiation enteritis	2	3.17%
Non-alcoholic fatty liver disease	2	3.17%
Crohn’s disease	2	3.17%
Total	63	100%

According to the total score of the baseline PSQI, the demographic characteristics of the two groups of patients were statistically analyzed, including gender, age, group size, and baseline quantitative table conditions. Due to different levels of patient compliance, not all patients had complete data. Therefore, the number of patients with different indicators in the short-term treatment group, medium-term treatment group, and long-term treatment group varied. The baseline conditions of patients in the sleep disorder group and the normal sleep group are shown in Table 2.

**Table 2:** Baseline conditions of patients in the sleep disorder group and the normal sleep group (Mean ± SD).

Demographic characteristic	Sleep disorder group (*n* = 40)	Normal sleep group (*n* = 23)	*p*
Age	45.45 ± 17.23	46.39 ± 13.76	0.824
Number of male (%)	17 (42.50)	18 (78.26)	0.151
**SF-36 health survey short form**			
Functioning (PF)	80.00 ± 16.76	89.57 ± 9.76	0.015
Role-physical (RP)	57.50 ± 45.71	60.87 ± 43.19	0.775
Bodily pain (BP)	74.50 ± 24.89	74.74 ± 19.37	0.969
General health (GH)	45.95 ± 21.04	46.78 ± 21.29	0.881
Vitality (VT)	56.25 ± 24.54	70.22 ± 16.34	0.018
Social functioning (SF)	61.45 ± 28.5	65.43 ± 20.60	0.560
Role-emotional (RE)	52.47 ± 46.49	60.87 ± 41.06	0.475
Mental health (MH)	52.50 ± 22.50	67.13 ± 16.67	0.009
**PSQI scale**			
Sleep quality (SQ)	1.90 ± 0.63	1.09 ± 0.60	<0.001
Sleep latency (SL)	2.38 ± 0.81	0.70 ± 0.70	<0.001
Sleep time (ST)	1.38 ± 1.10	0.30 ± 0.47	<0.001
Sleep efficiency (SE)	1.55 ± 1.13	0.30 ± 0.47	<0.001
Sleep disorders	2.48 ± 0.55	2.13 ± 0.46	0.014
Hypnotic drugs	1.00 ± 1.36	0.04 ± 0.21	0.001
Daytime dysfunction	0.95 ± 0.81	0.43 ± 0.59	0.010
PQSI total score	11.63 ± 2.86	5.09 ± 1.56	<0.001

### WMT improved sleep quality of sleep disorders patients

**Figure 2:**
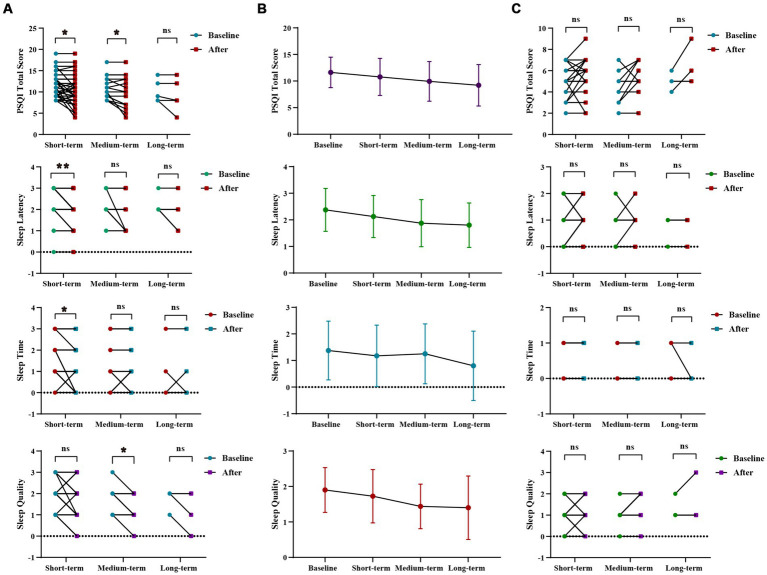
Changes of sleep quality after 1–3 times of WMT. **(A)** Comparison diagram of baseline and post-treatment in sleep disorder groups in SQ, SL, ST, PQSI total score (groups showing statistic difference) in different courses; **(B)** Line chat of baseline and post-treatment in sleep disorder groups in SQ, SL, ST, PQSI total score in different courses; **(C)** Comparison of baseline and post-treatment in normal sleep groups in SQ, SL, ST, PQSI total score in different courses. SQ, sleep quality; SL, sleep latency; ST, sleep time; PQSI, Pittsburgh Sleep Quality Index. * indicates *p* < 0.05; ** indicates *p* < 0.01; ns, not significant.

WMT had been shown to significantly improve sleep time, sleep latency, and the total score of the PSQI in the sleep disorder group during short-term treatment (*p* < 0.001 for sleep time, *p* = 0.031 for sleep latency, *p* = 0.026 for total score of PQSI) (Figures 2A,B; Supplementary Table S1). In the medium-term treatment, WMT improved sleep quality and the total PQSI score (*p* = 0.041 for sleep quality, *p* = 0.030 for PQSI) (Figures 2A,B; Supplementary Table S1). Possibly due to a lack of a sufficient sample size, there were no significant differences observed in the analysis of long-term treatment in the sleep disorder group. Of course, it was not excluded that psychological pressure, work and sleep environment, living habits and other factors affect the long-term improvement of WMT in patients with sleep disorders. However, it was worth noting that WMT treatment did not decrease sleep quality in individuals without sleep disorders (Figure 2C; Supplementary Table S1).

In sleep disorder group, there was no statistically significant difference in the WMT across different treatment courses. However, it was evident that the improvement in Sleep Quality and Sleep Duration increased with intensified treatment, demonstrating the superior efficacy of multiple courses over single or double courses. Furthermore, multiple and double courses showed a more substantial improvement in the PSQI total scores than a single course, suggesting the prioritization of multiple and double courses for efficient and stable enhancement of sleep quality (Supplementary Figure S1; Supplementary Table S2).

### WMT improved life quality of sleep disorders patients

**Figure 3:**
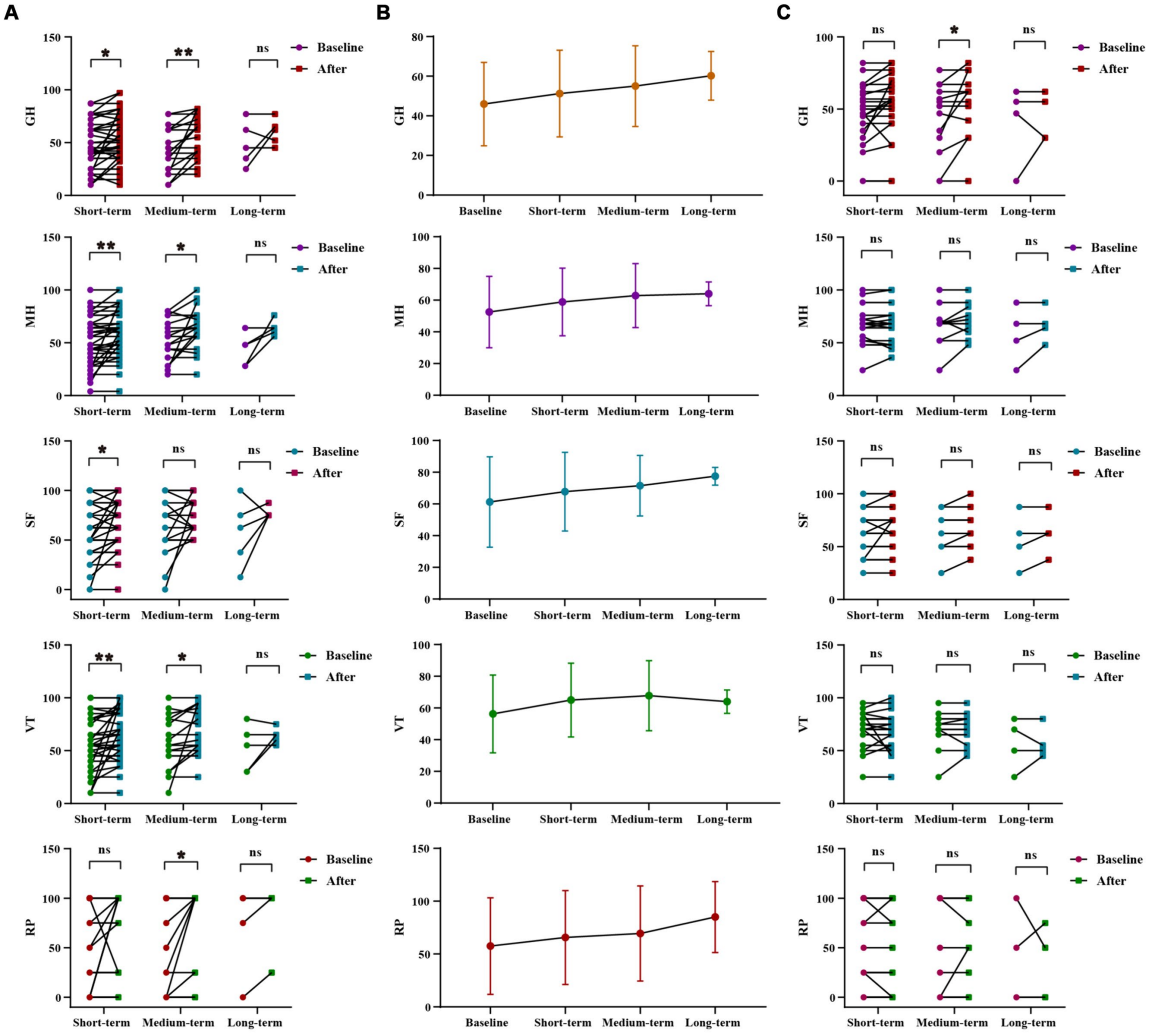
Changes of life quality after 1–3 times of WMT. **(A)** Comparison diagram of baseline and post-treatment in sleep disorder groups in RP, GH, VT, SF, MH (groups showing statistic difference) in different courses; **(B)** Line chat of baseline and post-treatment in sleep disorder groups in RP, GH, VT, SF, MH in different courses; **(C)** Comparison of baseline and post-treatment in normal sleep groups in RP, GH, VT, SF, MH in different courses. RP, role-physical; GH, general health; VT, vitality; SF, social function; MH, mental health. * indicates *p* < 0.05; ** indicates *p* < 0.01; ns, not significant.

WMT had been shown to significantly improve general health, vitality, social function and mental health in the sleep disorder group during short-term treatment (*p* = 0.013 for general health, *p* = 0.007 for vitality, *p* = 0.028 for social function, *p* = 0.006 for mental health). In the medium-term treatment, WMT significantly improved role-physical, general health, vitality and mental health (*p* = 0.045 for role-physical, *p* = 0.005 for general health, *p* = 0.046 for vitality, *p* = 0.0.16 for mental health). Possibly due to a lack of a sufficient sample size, there were no significant differences observed in the analysis of long-term treatment in the sleep disorder group. However, it was worth noting that WMT treatment did not decrease life quality in individuals without sleep disorders. WMT significantly improved general health in the medium-term (*p* = 0.041) (Figure 3; Supplementary Table S3).

Inter-group analysis of effective groups in sleep disorder group. In sleep disorder group, there was no statistically significant difference in the WMT across different treatment courses. However, it was evident that the improvement in vitality, social fiction and mental health increases with intensified treatment, demonstrating the superior efficacy of multiple courses over single or double courses. Furthermore, multiple and double courses showed a more substantial improvement in the role-physical and general health than a single course, suggesting the prioritization of multiple and double courses for efficient and stable enhancement of life quality and sleep quality (Supplementary Figures S2A,B; Supplementary Table S4).

Inter-group analysis of life quality improvement between sleep disorder and normal sleep group. The use of WMT improved the quality of life for patients with normal sleep. However, the degree of improvement in the quality of life was significantly higher for patients with sleep disorders compared to those with normal sleep. This difference was particularly notable in vitality (VT) and mental health (MH) (△VT, *p* = 0.029, △MH, *p* = 0.042), suggesting a more pronounced effect on the life quality of patients with sleep disorders (Supplementary Figure S2C; Supplementary Table S5).

### Correlation analysis between PQSI scale (sleep quality) and SF-36 scale (life quality)

Sleep quality (SQ) was strongly positively correlated with sleep latency (SL), the total score of PSQI (PSQI), physical functioning (RP), general health (GH), social functioning (SF), and mental health (MH). Sleep latency (SL) was strongly positively correlated with the total score of PSQI (PSQI), physical functioning (RP), general health (GH), vitality (VT), social functioning (SF), and mental health (MH). Sleep duration (ST) was strongly positively correlated with the total score of PSQI (PSQI), physical functioning (RP), and general health (GH). The total score of PSQI (PSQI) was strongly positively correlated with physical functioning (RP), general health (GH), social functioning (SF), and mental health (MH). Physical functioning (RP) was strongly positively correlated with general health (GH), vitality (VT), social functioning (SF), and mental health (MH). General health (GH) was strongly positively correlated with social functioning (SF) and mental health (MH). Vitality (VT) was strongly positively correlated with mental health (MH). Social functioning (SF) was strongly positively correlated with mental health (MH) (Supplementary Table S6).

### Safety analysis

Among the 164 instances of WMT treatment, there were a total of 3 adverse events (1.83%), mainly including diarrhea (2 cases, 1.22%) and anal pain (1 case, 0.61%). These adverse events were self-resolving or improved after symptomatic treatment, and no significant serious adverse events were observed in all patients. This suggested that WMT treatment had a good safety profile with a low incidence of adverse events.

### Analysis of gut microbiota composition before and after WMT

**Figure 4:**
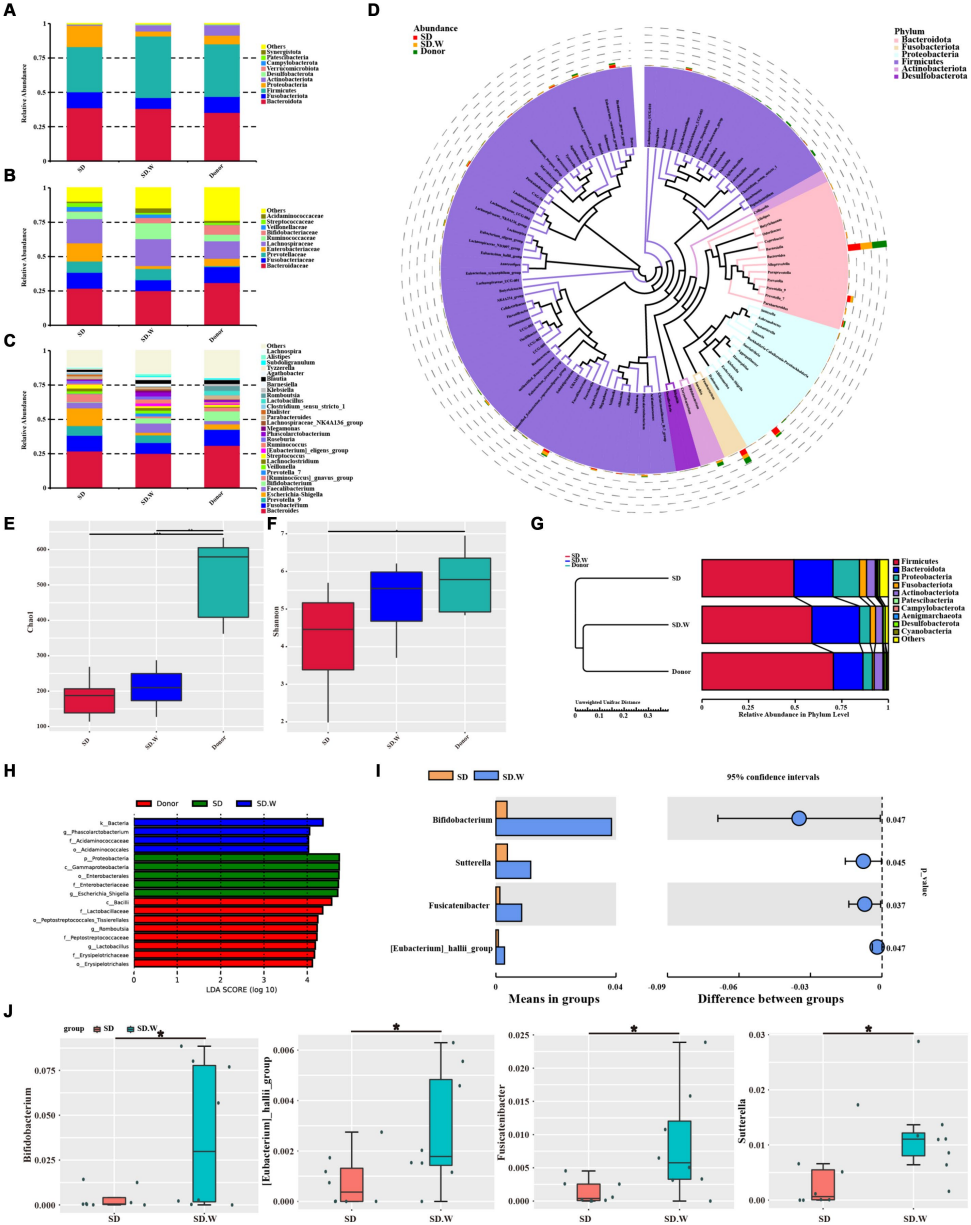
The composition of gut microbiota before and after WMT. **(A)** Composition of the top 10 gut microbiota at phylum level. **(B)** The composition of gut microbiota in the top 10 families. **(C)** The composition of gut microbiota in the top 30 genera. **(D)** Phylogenetic relationships of gut microbiota in the top 100 genera. **(E)** Chao1 index of α diversity analysis. **(F)** Shannon index of α diversity analysis. **(G)** β diversity analysis. **(H)** LEfSe analysis of SD group, SD. W group (SD group after WMT) and donor group. **(I,J)** *T*-test analysis before and after SD group WMT. * indicates *p* < 0.05; ** indicates *p* < 0.01.

We analyzed gut microbiota composition in the SD and donor groups before and after WMT. At the phylum level, the gut microbiota mainly included *Firmicutes*, *Bacteroidota*, *Fusobacteriota*, *Proteobacteria* and *Actinobacteriota*. At the phylum level, the relative abundance of *Firmicutes* and *Actinobacteriota* increased after WMT. The relative abundance of *Proteobacteria* and *Fusobacteriota* was decreased (Figure 4A). At the family level, the relative abundance of *Bifidobacteriaceae* and *Ruminococcaceae* increased after WMT. The relative abundance of *Enterobacteriaceae* and *Fusobacteriaceae* was reduced (Figure 4B). At the genus level, the relative abundance of *Bifidobacterium*, *Prevotella* 7, *[Ruminococcus] gnavus* group and *Faecalibacterium* was increased after WMT. The relative abundance of *Escherichia-Shigella* and *Streptococcus* was reduced (Figure 4C). We analyzed phylogenetic relationships at the genus level for the top 100 gut microbiota, The top six *Bacteroides*, *Fusobacterium, Prevotella* 9, *Escherichia-Shigella*, *Faecalibacterium* and *Bifidobacterium* were found (Figure 4D). Among them, WMT could not only increase the relative abundance of beneficial bacteria, such as *Bifidobacterium*, *Prevotella* 7, *[Ruminococcus] gnavus* group and *Faecalibacterium*, etc. In addition, it could reduce the relative abundance of harmful bacteria, such as *Escherichia-Shigella* and *Streptococcus* and so on.

WMT increased gut microbiota α diversity in the SD group, such as chao1 index and Shannon index (Figures 4E,F). The β diversity of gut microbiota after WMT was closer to that of the donor (Figure 4G). LEfSe analysis was performed on the SD group before and after WMT and the donor group to find the Biomarker with statistical difference between the groups. It was found that *Escherichia-Shigella* was the distinct species before WMT in SD group, while *Phascolarctobacterium* was the distinct species after WMT in SD group. The distinct species in the donor group was *Lactobacillus* and *Romboutsia* (Figure 4H). The species with significant differences between the SD group before and after WMT were identified by T-test. We found that WMT could significantly increase *Bifidobacterium*, *[Eubacterium] hallii* group, *Fusicatenibacter* and *Sutterella* in the SD group gut microbiota at the genus level of SD group gut microbiota (Figures 4I,J). It may be that WMT significantly increases these beneficial bacteria such as *Bifidobacterium* in the gut microbiota of SD patients, thus playing a role in improving sleep quality.

## Discussion

**Figure 5:**
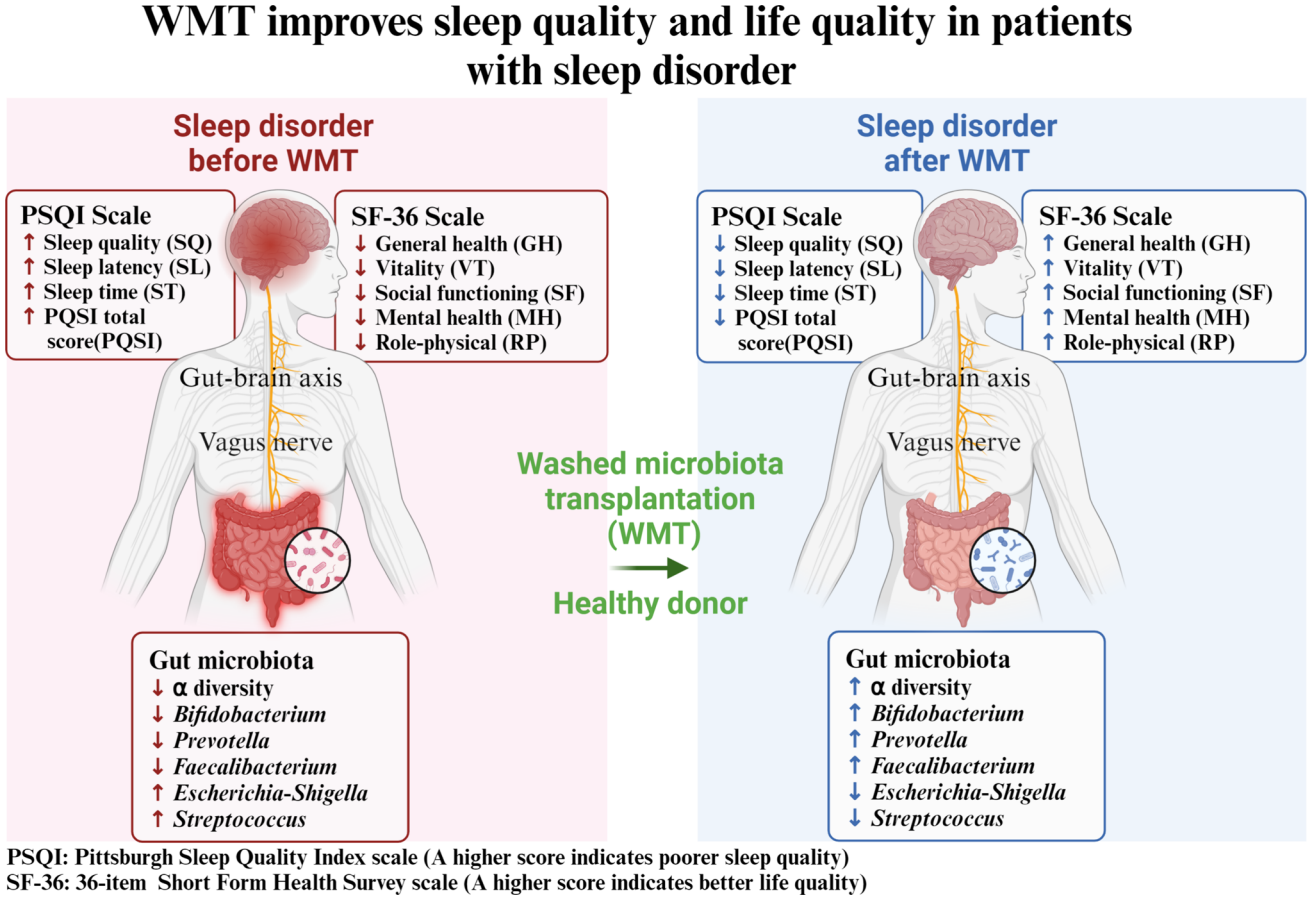
Washed microbiota transplantation improves sleep quality and life quality in patients with sleep disorder. PSQI: Pittsburgh Sleep Quality Index scale (A higher score indicates poorer sleep quality); SF-36: 36-item Short Form Health Survey scale (A higher score indicates better life quality).

In this study, we utilized a self-controlled before-and-after design to analyze the impact of WMT treatment on patients’ sleep quality and life quality (Figure 5). We assessed the changes in PSQI and SF-36 indicators after receiving the treatment. We sought to investigate if WMT treatment had any influence on the sleep quality of individuals without sleep abnormalities. Furthermore, we conducted an analysis of the gut microbiota in healthy donors to explore the potential of WMT as a means to replenish beneficial bacterial populations that could aid in improving sleep disorders. Our objective was to determine whether WMT treatment could effectively improve sleep quality and overall well-being in patients with sleep disorders. Our results showed that WMT significantly improved the sleep disorder patients in the short and medium term. In terms of sleep quality (SQ) and sleep latency (SL), the improvement value also increased with the increase of treatment course, and the improvement effect of multiple treatment course was better than that of single and double treatment course. In the total score (PSQI), the improvement effect of double and multiple treatment was better than that of single treatment. WMT also improved quality of life in the sleep disorder group. WMT regulated the disturbed gut microbiota in patients with sleep disorders. In the normal sleep group, WMT had no effect on the decline of sleep quality in the short, medium and long term, and had an improving effect on the quality of life.

FMT was the process of transferring extracted bacteria in the feces of healthy people to patient’s gut to regulate gut microbiota. The aim of FMT was to restore the healthy diversity of gut microbiota and provide effective treatment for certain diseases inside and outside the gut. Scientific studies indicated the potential role of microbiota in many diseases. The bidirectional communication between the microbiota and the gut-brain axis provided a theoretical basis for FMT to treat parenteral diseases (neurological disorders) (Parker et al., 2020). In animal models, FMT reduced the expression of NOD-like receptor protein 3 in the brain caused by chronic and unpredictable mild stress, thereby inhibiting depression-like behavior (Rao et al., 2021). In addition, FMT in mice exposed to chronic intermittent hypoxia causes sleep–wake disorders and sleep disturbances in mice (Badran et al., 2020). Interestingly, in clinical studies of FMT in the treatment of IBS, FMT from healthy donors improved sleep status and the Hamilton Depression Scale (Kurokawa et al., 2018). This suggested that using FMT to reshape the receptor gut microbiota could be a future approach to treating central nervous system disorders, such as sleep disorders. This result was consistent with our research, which showed that WMT significantly improved the sleep disorder patients in the short and medium term. In terms of sleep quality (SQ) and sleep latency (SL), the improvement value also increased with the increase of treatment course, and the improvement effect of multiple treatment course was better than that of single and double treatment course. In the total score (PSQI), the improvement effect of double and multiple treatment was better than that of single treatment.

In studies on the quality of life of patients with Parkinson’s disease, it was found that the decline of sleep quality and mood disorders were important influencing components, and the two were closely related (Palmeri et al., 2019). In animal experiments, gut microbiota had a certain impact on the development of anxiety and depression, and the tested mice also showed symptoms related to depression after transplantation of fecal microbiota of mice with negative behaviors (Sun et al., 2018). Pearson-Leary et al. (2020) found that FMT could increase the levels of dopamine and 5-HT in the striatum of mice. Then, it was speculated that the reconstruction of intestinal microecology could change the levels of dopamine, 5-HT and other neurotransmitters in the body, thereby improving the symptoms of mood disorders. In addition, studies had found that patients with symptoms of stress and anxiety were less than before after receiving treatment with specific lactobacillus strains (Lew et al., 2019). Our results showed that WMT can not only increase the relative abundance of beneficial bacteria, such as *Bifidobacterium*, *Prevotella* 7, *[Ruminococcus] gnavus* group, and *Faecalibacterium*, etc. In addition, it could reduce the relative abundance of harmful bacteria, such as *Escherichia-Shigella* and *Streptococcus* and so on. As Misra summarized in the article, current strains of psychoprobiotics were used for anxiety, depression, and stress (Misra and Mohanty, 2019). He could find that current psychoprobiotics were mainly concentrated in *Lactobacillus* and *Bifidobacterium*, and different strains had their own specific mental health benefits. Perhaps it was WMT that could increase the type and abundance of psychoprobiotics and reduce harmful bacteria, so as to play a role in alleviating sleep disorders. The clinical symptoms of mood disorders such as anxiety and depression could be manifested as decreased sleep quality (Rana et al., 2018; Chang et al., 2019). From the perspective of intestinal microbial metabolism, the level of 5-HT in the host body could regulate brain activity and thus affect sleep (Ge et al., 2018). In addition, the assurance of sleep quality was crucial to the metabolism of the central nervous system, especially in the removal of metabolic waste (Xie et al., 2013). Lack of sleep could have an impact on cognition and memory ability (Maquet, 2001), accompanied by the decline of spatial cognitive positioning ability (Wu et al., 2013), increased pain (Vanini, 2016), decreased immunity (Wilder-Smith et al., 2013), and disorder of stress level (Voderholzer et al., 2004). It also increased the risk of Alzheimer’s disease (Liguori et al., 2020), inflammatory bowel disease (Wu et al., 2020), non-alcoholic fatty liver disease (Wei et al., 2020), obesity (de Baca et al., 2019), diabetes (Brouwer et al., 2020), cardiovascular events (Sekine et al., 2010) and other diseases. Studies had found that the behavioral damage caused by chronic sleep disorders could be alleviated by regulating gut microbiota and supplementing probiotics (Dhaliwal et al., 2018), and the cognitive function of rats had been improved compared with before (Yang et al., 2018), which was observed to be accompanied by a decline in the level of interleukin-6. In our study, WMT not only improved sleep quality, but also improved quality of life in the sleep disorder group. WMT significantly improved general health (GH), vitality (VT), social function (SF) and mental health (MH) in the short term. WMT significantly improved physiological function (RP), general health (GH), vitality (VT) and mental health (MH) in the medium term. We had also observed that WMT modulated the disturbed gut microbiota of patients with sleep disorders and played a role in alleviating sleep disorders. However, we did not analyze the material basis of this effect through metabolomics techniques, which required further exploration.

At present, the safety of WMT therapy was the focus of clinical work. In a study on the application of WMT to gastroesophageal reflux disease, it was observed that only 1 out of 15 patients undergoing WMT showed mild adverse events, manifested as mushy stool 2–3 times a day, which could be resolved on its own, except for no other serious adverse events related to WMT treatment (Zheng et al., 2021). In a study of children aged 3–7 years who received scrubber microflora transplantation (Zhong et al., 2021), no adverse events of mild to severe abdominal pain or diarrhea were observed during the injection of scrubber microflora or drug suspension through colonic catheterization. However, it should be noted that 4 parents (8.51%) complained that the catheter retention had a significant impact on their children’s activities during the treatment period. They had difficulty tolerating the catheterization, and this discomfort was largely due to the patient’s youth, with no other serious adverse events reported. In our study of WMT treatment, we found that the incidence of adverse events after WMT was low (1.83%). The main adverse events were diarrhea and anal discomfort. No serious adverse events were observed in this study, suggesting that WMT may be a safe treatment to improve patients’ quality of life and sleep quality. We established clinical evidence of the effects of WMT on SD, which laid a foundation for subsequent studies on the effects of environmental factors (Wu et al., 2021a), gut microbiota (Wu et al., 2021b, 2022a,b,c) and metabolic biomarkers (Wu et al., 2021c, 2023) on SD. Our follow-up clinical studies suggested WMT frequency was a key factor to the efficacy of SD patients. Altogether, these results suggested the beneficial effect of the WMT in SD, but its mechanism need additional investigation.

There were some limitations to our study. First of all, our total sample size was a little small. The number of people in the study who were able to receive long-term treatment for WMT was even smaller. Therefore, more data were needed to confirm the long-term efficacy of WMT in the treatment of SD. Secondly, our study mainly focused on the analysis of Sleep quality and life quality, the metabolomics of gut before and after WMT had not been evaluated. Therefore, the related metabolite of WMT on SD remained unclear. Third, we did not evaluate the confusion factors between the main diseases for WMT treatment and SD. Although our study showed that WMT could improve SD in both short and medium term, large-scale prospective studies were needed to further approved our conclusions. In the future, we will keep follow-up all the patients of this study, and plan to conduct a large sample prospective study to verify the effect of WMT on SD.

## Conclusion

WMT could significantly improve sleep quality in patients with sleep disorders with no adverse events. The improvement in sleep quality resulting from WMT could lead to an overall enhancement in life quality. WMT could be a potentially effective treatment for patients with sleep disorders by regulating the gut microbiota homeostasis.

## Data availability statement

The datasets presented in this study can be found in online repositories. The data presented in the study are deposited in the NCBI Sequence Read Archive repository, accession number PRJNA1104761, available at: https://www.ncbi.nlm.nih.gov/sra/PRJNA1104761.

## Ethics statement

The studies involving humans were approved by Ethics Committee (No. 2017-98) in accordance with the Declaration of Helsinki at the First Affiliated Hospital of Guangdong Pharmaceutical University, Guangzhou, China. The studies were conducted in accordance with the local legislation and institutional requirements. The participants provided their written informed consent to participate in this study.

## Author contributions

HH: Conceptualization, Formal analysis, Investigation, Validation, Writing – original draft. ML: Conceptualization, Formal analysis, Methodology, Validation, Writing – original draft. YQ: Conceptualization, Methodology, Validation, Writing – original draft. ZW: Conceptualization, Methodology, Validation, Writing – original draft. LW: Funding acquisition, Resources, Supervision, Writing – review & editing.

## Footnotes

^1^ http://ccb.jhu.edu/software/FLASH/
