# Supplementary material for: Washed microbiota transplantation improves sleep quality in patients with sleep disorder by the gut-brain axis
Source: Front Neurosci. 2024 Jun 24;18:1415167. doi: 10.3389/fnins.2024.1415167 (PMC11228149; doi:10.3389/fnins.2024.1415167)
Supplement: Supplementary file 1 [file Data_Sheet_1.docx]

**Supplumentary Table 1 Comparison of Baseline and Post-Treatment PSQI Scale Status in Patients**

| Dimensions | Baseline  (n=40) | Short-term  (n=40) | P value | Baseline  (n=16) | Medium-term  (n=16) | P value | Baseline  (n=5) | Long-term  (n=5) | P value |
| --- | --- | --- | --- | --- | --- | --- | --- | --- | --- |
| Sleep disorder group | | | | | | | | | |
| Sleep quality | 1.90 ± 0.63 | 1.73±0.75 | 0.700 | 1.89±0.60 | 1.44±0.63 | 0.041 | 1.80±0.45 | 1.40±0.89 | 0.178 |
| Sleep latency | 2.38 ± 0.81 | 2.13±0.79 | 0.001 | 2.19±0.75 | 1.88±0.88 | 0.055 | 2.20±0.45 | 1.80±0.84 | 0.178 |
| Sleep time | 1.38 ± 1.10 | 1.18±1.15 | 0.031 | 1.19±1.17 | 1.25±1.13 | 0.669 | 0.80±1.30 | 0.80±1.30 | ＞0.999 |
| Sleep efficiency | 1.55 ± 1.13 | 1.55±1.11 | ＞0.999 | 1.38±1.20 | 1.31±1.20 | 0.580 | 1.02±1.30 | 1.02±1.30 | ＞0.999 |
| Sleep disorders | 2.48 ± 0.55 | 2.45±0.55 | 0.711 | 2.56±0.51 | 2.56±0.50 | ＞0.999 | 2.80±0.45 | 2.60±0.55 | 0.374 |
| Hypnotic drugs | 1.00 ± 1.36 | 0.90±1.26 | 0.352 | 1.00±1.41 | 0.75±0.18 | 0.300 | 0.80±1.30 | 0.80±1.30 | ＞0.999 |
| Daytime dysfunction | 0.95 ± 0.81 | 0.85±0.74 | 0.291 | 1.00±0.89 | 0.75±0.68 | 0.164 | 1.00±0.71 | 1.00±0.71 | ＞0.999 |
| PQSI total score | 11.63 ± 2.86 | 10.78±3.48 | 0.016 | 11.00±2.63 | 9.94±3.73 | 0.030 | 10.20±2.68 | 9.20±3.90 | 0.266 |
| Dimensions | Baseline  (n =23) | Short-term  (n=23) | P value | Baseline  (n=10) | Medium-term  (n=10) | P value | Baseline  (n=4) | Long-term  (n=4) | P value |
| Normal sleep group | | | | | | | | | |
| Sleep quality | 1.09 ± 0.60 | 1.13±0.69 | 0.714 | 1.00±0.47 | 1.30±0.67 | 0.081 | 1.25±0.50 | 1.50±1.00 | 0.391 |
| Sleep latency | 0.70 ± 0.70 | 0.83±0.65 | 0.266 | 0.60±0.70 | 0.70±0.67 | 0.591 | 0.75±0.50 | 0.75±0.50 | ＞0.999 |
| Sleep time | 0.30± 0.47 | 0.30±0.47 | ＞0.999 | 0.20±0.42 | 0.20±0.42 | ＞0.999 | 0.50±0.58 | 0.25±0.50 | 0.391 |
| Sleep efficiency | 0.30 ± 0.47 | 0.35±0.57 | 0.575 | 0.20±0.42 | 0.10±0.32 | 0.343 | 0.25±0.50 | 1.00±0.82 | 0.215 |
| Sleep disorders | 2.13± 0.46 | 2.22±0.52 | 0.539 | 2.10±0.57 | 2.40±0.52 | 0.193 | 3.25±1.89 | 2.50±0.58 | 0.391 |
| Hypnotic drugs | 0.04 ± 0.21 | 0.00±0.00 | 0.328 | 0.00±0.00 | 0.00±0.00 | ＞0.999 | 0.00±0.00 | 0.00±0.00 | ＞0.999 |
| Daytime dysfunction | 0.43 ± 0.59 | 0.52±0.67 | 0.492 | 0.30±0.67 | 0.60±0.84 | 0.193 | 0.00±0.00 | 0.25±0.50 | 0.391 |
| PQSI total score | 5.09 ± 1.56 | 5.35±1.72 | 0.408 | 4.00±1.56 | 5.30±1.57 | 0.108 | 4.00±1.41 | 6.25±1.89 | 0.265 |

**Supplumentary Table 2: Comparison of score improvement in SQ, SL, ST, PQSI total score in different courses**

| Improvement value | Short-term(n=40) | medium-term(n =16) | long-term(n =5) | P value |
| --- | --- | --- | --- | --- |
| △Sleep quality | 0.18 ± 0.59 | 0.25 ± 0.45 | 0.40 ± 0.55 | 0.665 |
| △Sleep latency | 0.25 ± 0.44 | 0.31 ± 0.60 | 0.40 ± 0.55 | 0.775 |
| △Sleep time | 0.20 ± 0.56 | -0.06 ± 0.57 | 0.00 ± 0.71 | 0.283 |
| △PSQI total score | 0.85 ± 2.14 | 1.06 ± 1.77 | 1.00 ± 1.73 | 0.935 |

Note: △means the variation of this group before and after different courses of treatment. Data are presented by means±standard deviation, and P value less than 0.05 is considered as statistic difference.

**Supplumentary Table 3 Comparison of Baseline and Post-Treatment SF-36 Scale Status in Patients**

|  | **Baseline**  (n=40) | **Short-term**  (n=40) | **P value** | **Baseline**  (n=18) | **Medim-term**  (n=18) | **P value** | **Baseline**  (n=5) | **Long-term**  (n=5) | **P value** |
| --- | --- | --- | --- | --- | --- | --- | --- | --- | --- |
| **Sleep disorder group** | | | | | | | | | |
| PF | 80.00 ± 16.76 | 81.50±16.22 | 0.327 | 78.89±18.83 | 78.61±17.64 | 0.826 | 83.00±16.81 | 87.00±5.70 | 0.495 |
| RP | 57.50 ± 45.71 | 65.63±44.47 | 0.102 | 54.17±46.38 | 69.44±45.01 | 0.045 | 75.00±43.30 | 85.00±33.54 | 0.178 |
| BP | 74.50 ± 24.89 | 77.55±16.51 | 0.372 | 72.06±27.92 | 81.00±12.66 | 0.174 | 71.60±24.18 | 77.20±6.72 | 0.615 |
| GH | 45.95 ± 21.04 | 51.25±21.86 | 0.013 | 42.33±21.76 | 55.00±20.39 | 0.005 | 48.80±20.86 | 60.20±12.32 | 0.286 |
| VT | 56.25 ± 24.54 | 65.00±23.29 | 0.007 | 57.22±24.57 | 67.78±22.11 | 0.046 | 52.00±21.97 | 64.00±7.42 | 0.229 |
| SF | 61.45 ± 28.56 | 68.00±24.83 | 0.028 | 61.28±29.00 | 71.78±19.11 | 0.116 | 57.80±33.55 | 77.60±5.81 | 0.243 |
| RE | 52.47 ± 46.49 | 55.82±44.93 | 0.485 | 61.11±44.68 | 63.00±44.14 | 0.579 | 66.60±47.20 | 93.40±14.76 | 0.178 |
| MH | 52.50 ± 22.50 | 58.90±21.32 | 0.006 | 52.00±19.69 | 62.89±20.16 | 0.016 | 43.20±15.34 | 64.00±7.48 | 0.063 |
|  | **Baseline**  (n =23) | **Short-term**  (n=23) | **P value** | **Baseline**  (n =13) | **Medim-term**(n=13) | **P value** | **baseline**(n=4) | **Long-term**  (n=4) | **P value** |
| **Normal sleep group** | | | | | | | | | |
| PF | 89.57± 9.76 | 90.43±9.76 | 0.357 | 88.08± 8.79 | 90.38±8.03 | 0.213 | 88.75±6.29 | 85.00±13.54 | 0.391 |
| RP | 60.87 ± 43.19 | 59.78±44.43 | 0.575 | 51.92± 48.37 | 53.85±44.31 | 0.673 | 57.50±47.87 | 51.25±37.50 | 0.718 |
| BP | 74.74 ± 19.37 | 78.13±17.05 | 0.385 | 77.78± 19.84 | 75.85±24.56 | 0.799 | 77.75±5.50 | 80.50±6.35 | 0.391 |
| GH | 46.78 ± 21.29 | 51.65±22.44 | 0.053 | 43.77± 25.10 | 53.69±23.13 | 0.041 | 41.00±28.01 | 44.25±16.70 | 0.761 |
| VT | 70.22 ± 16.34 | 69.13±17.10 | 0.630 | 69.62± 17.26 | 70.38±14.36 | 0.711 | 56.25±24.28 | 57.50±15.55 | 0.873 |
| SF | 65.43 ± 20.60 | 67.57±20.51 | 0.257 | 65.54± 19.34 | 68.46±18.12 | 0.082 | 56.50±26.26 | 63.00±20.41 | 0.182 |
| RE | 60.87 ± 41.06 | 59.43±40.20 | 0.575 | 69.23± 39.63 | 71.77±40.52 | 0.725 | 50.00±43.12 | 50.00±43.12 | ＞0.999 |
| MH | 67.13 ± 16.67 | 67.30±16.61 | 0.847 | 68.00± 18.90 | 72.31±14.92 | 0.110 | 58.00±27.03 | 67.00±16.45 | 0.215 |

Note: Data are presented by means±standard deviation, and P value less than 0.05 is considered as statistic difference. PF means physical fiction, RP means role-physical, BP means body pain, GH means genera health, VT means vitality, SF means social function, RE means role-emotional, MH means mental health.

**Supplumentary Table 4 Comparison of score improvement in RP, GH, VT, SF, MH in different courses.**

| Improvement value | Short-term(n=40) | medium-term(n=18) | long-term(n=5) | P value |
| --- | --- | --- | --- | --- |
| △RP | 8.13 ± 30.69 | 15.28 ± 29.88 | 10.00 ± 13.69 | 0.843 |
| △GH | 5.30 ± 12.87 | 12.67 ± 16.85 | 11.40 ± 20.73 | 0.483 |
| △VT | 8.75 ± 19.47 | 10.56 ± 20.86 | 12.00 ± 18.91 | 0.924 |
| △SF | 6.56 ± 18.12 | 10.42 ± 26.86 | 20.00 ± 32.60 | 0.345 |
| △MH | 6.40 ± 14.00 | 10.89 ± 17.24 | 20.80 ± 18.20 | 0.083 |

Note: △means the variation of this group before and after different courses of treatment. Data are presented by means±standard deviation, and P value less than 0.05 is considered as statistic difference.

**Supplumentary Table 5 Comparison of life quality improvement in different courses.**

| **Improvement value** | **Short-term** | | | **Medium-term** | | | **Long-term** | | |
| --- | --- | --- | --- | --- | --- | --- | --- | --- | --- |
|  | Sleep disorder group(n=40) | Normal sleep group(n=23) | P value | Sleep disorder group(n=18) | Normal sleep group(n=13) | P value | Sleep disorder group(n=5) | Normal sleep group(n=4) | P value |
| △PF | 1.50 ± 9.55 | 0.87 ± 4.43 | 0.767 | -0.28 ± 5.28 | 2.31 ± 6.33 | 0.226 | 4.00 ± 11.94 | -3.75 ± 7.50 | 0.298 |
| △RP | 8.13 ± 30.69 | -1.09 ± 9.16 | 0.167 | 15.28 ± 29.88 | 1.92 ± 16.01 | 0.154 | 10.00 ± 13.69 | -6.25 ± 31.46 | 0.328 |
| △BP | 3.06 ± 21.38 | 3.38 ± 18.31 | 0.951 | 8.95 ± 26.75 | -1.92 ± 26.56 | 0.272 | 5.56 ± 22.99 | 2.78 ± 5.56 | 0.822 |
| △GH | 5.30 ± 12.87 | 4.87 ± 11.41 | 0.895 | 12.67 ± 16.85 | 9.92 ± 15.63 | 0.648 | 11.40 ± 20.73 | 3.25 ± 19.55 | 0.567 |
| △VT | 8.75 ± 19.47 | -1.09 ± 10.66 | 0.029 | 10.56 ± 20.86 | 0.77 ± 7.32 | 0.117 | 12.00 ± 18.91 | 1.25 ± 14.36 | 0.380 |
| △SF | 6.56 ± 18.12 | 2.17 ± 8.96 | 0.282 | 10.42 ± 26.86 | 2.88 ± 5.48 | 0.330 | 20.00 ± 32.60 | 6.25 ± 7.22 | 0.441 |
| △RE | 3.33 ± 30.00 | -1.45 ± 12.22 | 0.469 | 1.85 ± 13.87 | 2.56 ± 25.32 | 0.921 | 26.67 ± 36.51 | 0.00 ± 66.67 | 0.467 |
| △MH | 6.40 ± 14.00 | 0.17 ± 4.26 | 0.042 | 10.89 ± 17.24 | 4.31 ± 9.01 | 0.220 | 20.80 ± 18.20 | 9.00 ± 11.49 | 0.299 |

**Supplumentary Table 6 Correlation analysis between PQSI scale and SF-36 scale.**

| Sleep quality（SQ）-sleep latency(SL) | correlation | 0.994 |
| --- | --- | --- |
|  | p value | 0.006 |
| Sleep quality（SQ）-sleep time(ST) | correlation | 0.702 |
|  | p value | 0.298 |
| Sleep quality（SQ）-PQSI TOTAL SCORE(PSQI) | correlation | 0.973 |
|  | p value | 0.027 |
| Sleep quality（SQ）-role-physical（RP） | correlation | -0.867 |
|  | p value | 0.113 |
| Sleep quality（SQ）-general health（GH） | correlation | -0.953 |
|  | p value | 0.047 |
| Sleep quality（SQ）-vitality（VT） | correlation | -0.782 |
|  | p value | 0.218 |
| Sleep quality（SQ）-social function（SF） | correlation | -0.947 |
|  | p value | 0.053 |
| Sleep quality（SQ）-mental Health（MH） | correlation | -0.975 |
|  | p value | 0.025 |
| Sleep latency(SL)-sleep time(ST) | correlation | 0.749 |
|  | p value | 0.251 |
| Sleep latency(SL)-PQSI TOTAL SCORE(PSQI) | correlation | 0.984 |
|  | p value | 0.016 |
| Sleep latency(SL)-role-physical（RP） | correlation | -0.892 |
|  | p value | 0.108 |
| Sleep latency(SL)-general health（GH） | correlation | -0.971 |
|  | p value | 0.029 |
| Sleep latency(SL)-vitality（VT） | correlation | -0.808 |
|  | p value | 0.192 |
| Sleep latency(SL)）-social function（SF） | correlation | -0.969 |
|  | p value | 0.031 |
| Sleep latency(SL)-mental health（MH） | correlation | -0.992 |
|  | p value | 0.008 |
| Sleep time(ST)-PQSI TOTAL SCORE(PSQI) | correlation | 0.847 |
|  | p value | 0.153 |
| Sleep time(ST)-role-physical（RP） | correlation | -0.961 |
|  | p value | 0.039 |
| Sleep time(ST)-general health（GH） | correlation | -0.884 |
|  | p value | 0.116 |
| Sleep time(ST)-vitality（VT） | correlation | -0.366 |
|  | p value | 0.634 |
| Sleep time(ST)-social function（SF） | correlation | -0.889 |
|  | p value | 0.111 |
| Sleep time(ST)-mental health（MH） | correlation | -0.732 |
|  | p value | 0.268 |
| PQSI TOTAL SCORE(PSQI)-role-physical（RP） | correlation | -0.958 |
|  | p value | 0.042 |
| PQSI TOTAL SCORE(PSQI)-general health（GH） | correlation | -0.997 |
|  | p value | 0.003 |
| PQSI TOTAL SCORE(PSQI)-vitality（VT） | correlation | -0.703 |
|  | p value | 0.297 |
| PQSI TOTAL SCORE(PSQI)-social function（SF） | correlation | -0.994 |
|  | p value | 0.006 |
| PQSI TOTAL SCORE(PSQI)-mental health（MH） | correlation | -0.965 |
|  | p value | 0.035 |
| Role-physical（RP）-general health（GH） | correlation | 0.975 |
|  | p value | 0.025 |
| Role-physical（RP）-vitality（VT） | correlation | 0.519 |
|  | p value | 0.481 |
| Role-physical（RP）-social function（SF） | correlation | 0.974 |
|  | p value | 0.026 |
| Role-physical（RP）-mental health（MH） | correlation | 0.867 |
|  | p value | 0.133 |
| General health（GH）-vitality（VT） | correlation | 0.682 |
|  | p value | 0.318 |
| General health（GH）-social function（SF） | correlation | 0.999 |
|  | p value | 0.001 |
| General health（GH）-mental health（MH） | correlation | 0.954 |
|  | p value | 0.046 |
| Gitality（VT）-social function（SF） | correlation | 0.694 |
|  | p value | 0.306 |
| Gitality（VT）-mental health（MH） | correlation | 0.866 |
|  | p value | 0.134 |
| Social function（SF）-mental health（MH） | correlation | 0.957 |
|  | p value | 0.043 |

Note: **|r| > 0.8**
