# Supplementary material for: Washed microbiota transplantation improves sleep quality in patients with sleep disorder by the gut-brain axis
Source: Front Neurosci. 2024 Jun 24;18:1415167. doi: 10.3389/fnins.2024.1415167 (PMC11228149; doi:10.3389/fnins.2024.1415167)
Supplement: Supplementary file 2 [file Data_Sheet_2.pdf]

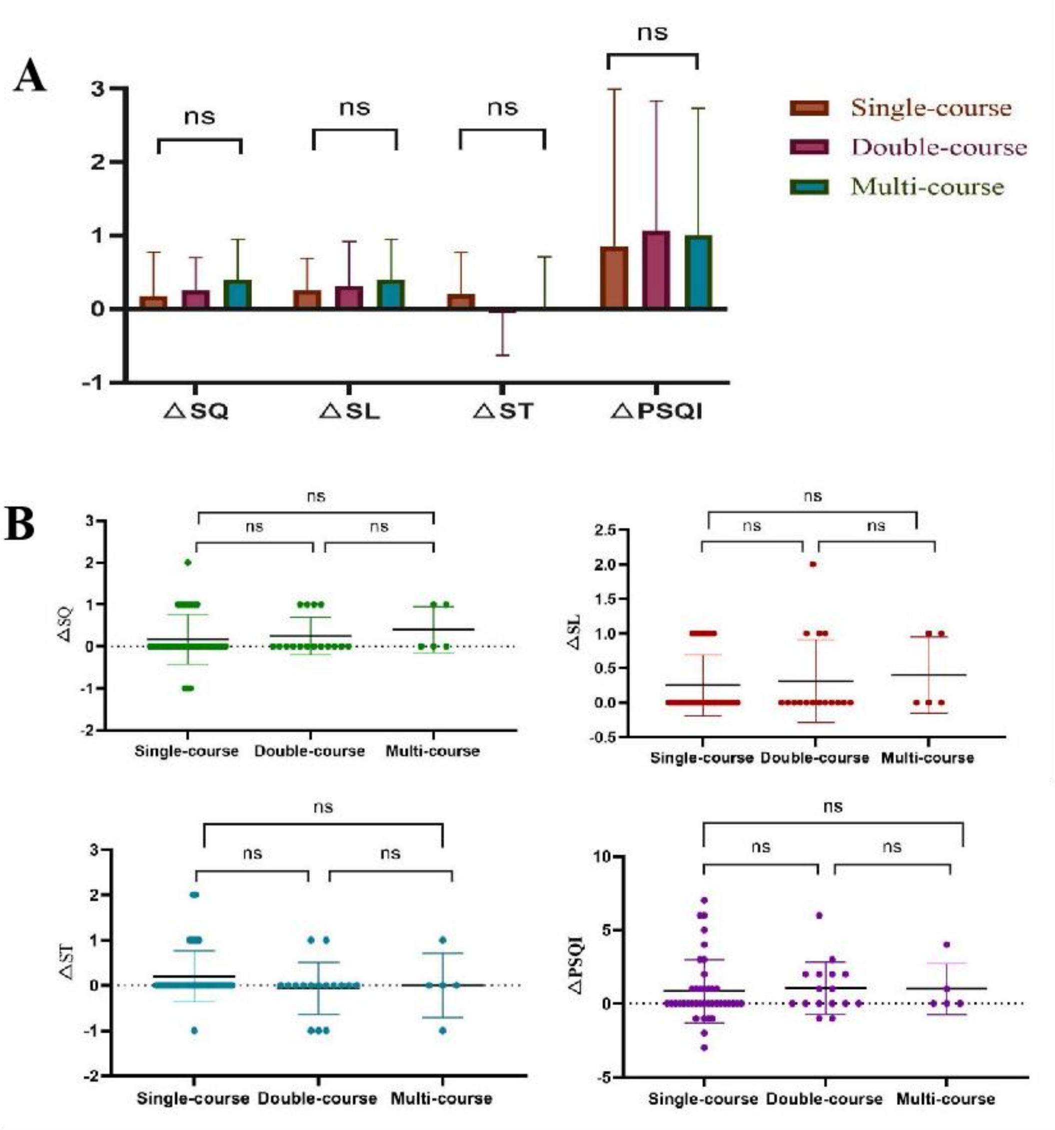

Supplumentary Figure 1 Analysis of inter-group differences in sleep quality changes. (A) Histogram of improvement in SQ, SL, ST, PQSI total score in different courses; (B) Intergroup analysis of improvement in SQ, SL, ST, PQSI total score in different courses. ns, not significant.

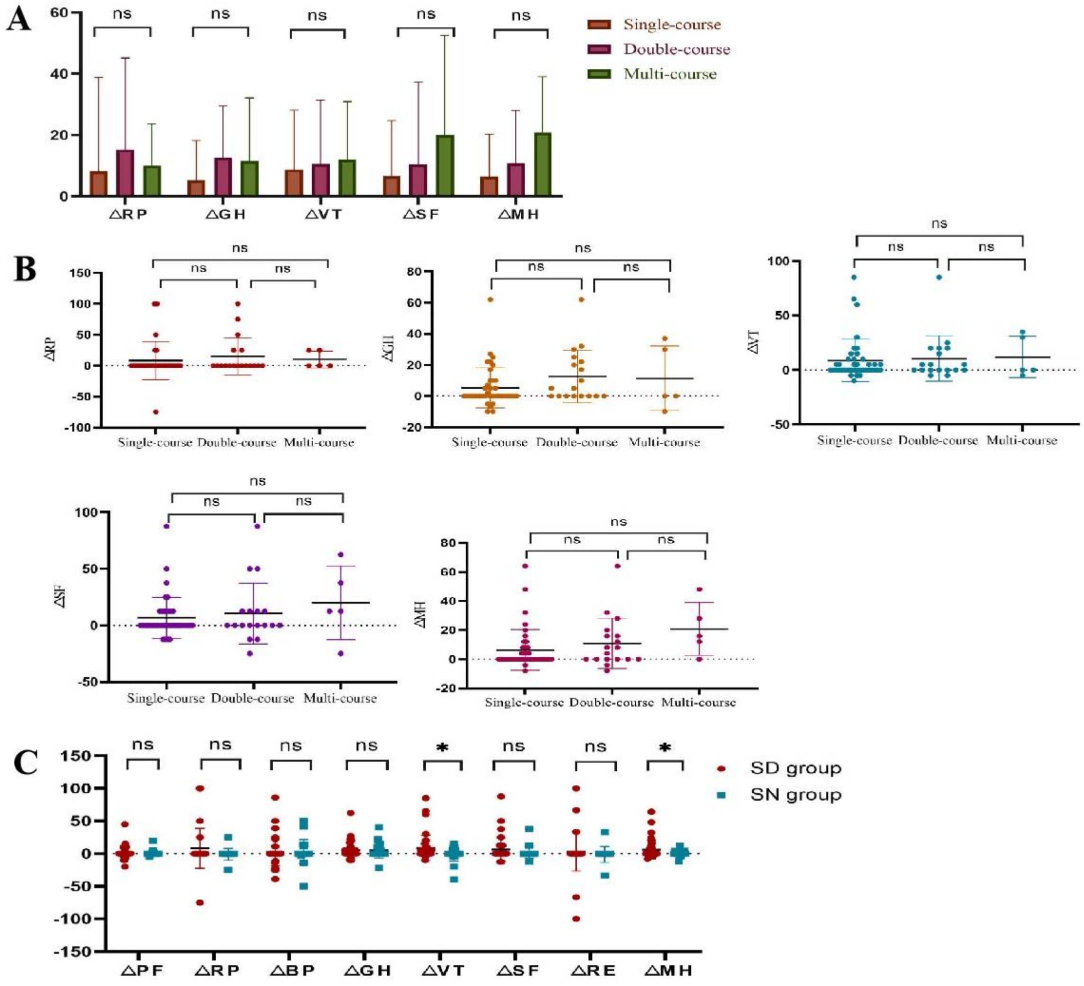

Supplumentary Figure 2 Analysis of inter-group differences in life quality changes. (A) Histogram of improvement in RP, GH, VT, SF, MH in different courses; (B) Intergroup analysis of improvement in RP, GH, VT, SF, MH in different courses. ns, not significant; (C) Histogram of life quality improvement in short-term group. ns, not significant.
